# Alternative splicing isoform in succinate dehydrogenase complex, subunit C causes downregulation of succinate-coenzyme Q oxidoreductase activity in mitochondria

**DOI:** 10.3892/ol.2014.2699

**Published:** 2014-11-11

**Authors:** NANA SATOH, CHIKAKO YOKOYAMA, NORIAKI ITAMURA, YOSHIHARU MIYAJIMA-NAKANO, HISASHI HISATOMI

**Affiliations:** Laboratory of Cellular and Molecular Biochemistry, Department of Materials and Life Science, Seikei Universty, Musashino, Tokyo 180-8633, Japan

**Keywords:** succinate dehydrogenase complex, subunit C, alternative splicing, dominant-negative inhibitor

## Abstract

Mitochondrial succinate dehydrogenase (SDH) is localized to the inner mitochondrial membrane and is responsible for the redox of succinic acid. SDH is a tetrameric iron-sulfur flavoprotein of the tricarboxylic acid cycle and respiratory chain. The SDH complex, subunit C (*SDHC*) transcript has deletion-type alternative splicing sites. Generally, alternative splicing produces variant proteins and expression patterns, as products of different genes. In certain cases, specific alternative splicing variants (ASVs) have been associated with human disease. Due to a frameshift mutation causing loss of the heme binding region, the *SDHC* Δ5 isoform (lacking exon 5) exhibits no SDHC activity. To investigate whether the *SDHC* splicing variants can function as dominant-negative inhibitors, *SDHC* ASVs were overexpressed in HCT-15 human colorectal cancer cells. Using real-time reverse transcription-polymerase chain reaction, a dominant-negative effect of the Δ5 isoform on *SDHC* mRNA was shown. In addition, Δ5 overexpression increased the levels of reactive oxygen species. Furthermore, in the Δ5 isoform-overexpressing cells, SDH activity was reduced. SDHC activation is a significant event during the electron transport chain, and the function of the *SDHC* Δ5 variant may be significant for the differentiation of tumor cells.

## Introduction

Mitochondrial succinate dehydrogenase (SDH) is a tetrameric iron-sulfur flavoprotein of the tricarboxylic acid (TCA) cycle and respiratory chain. The SDH complex, subunit C (SDHC) is a membrane-anchoring subunit of SDH that is tethered to the mitochondrial matrix (1). SDH oxidizes succinate to fumarate in the eighth step of the TCA respiratory cycle (2), generating two hydrogen ions and two electrons, which are transferred to ubiquinone through the iron-sulfur cluster and flavin adenine dinucleotide. SDHC is involved in the TCA cycle and electron transport chain (3–5).

Hereditary paragangliomas (PGLs) are neuroendocrine tissue tumors that arise symmetrically along the spinal axis between the skull and the pelvis (6). The pathogenic mechanism of PGLs has not yet been elucidated; however, mRNA splicing deficiencies of proteins lacking normal function have been implicated in various diseases, including PGLs (7–9). Alternative splicing of primary transcripts is a fundamental biological process involved in gene expression. In general, alternative splicing produces variant proteins and expression patterns as products of different genes. Up to one-third of human genes are alternatively spliced (10), and several alternative splicing variants (ASVs) have been associated with human diseases (11,12). The human *SDHC* gene maps to q23.3 on the long arm of chromosome 1, spanning >50 kb, and is composed of seven exons separated by intronic sequences (13). The complete cDNA sequence encompasses full-length *SDHC*, encoding a 169-amino acid (aa) polypeptide. Deleted *SDHC* transcripts have been characterized, comprising two isoforms generated by in-frame deletions of 102 nucleotides corresponding to the complete loss of exon 3, and a frameshift deletion of 164 nucleotides corresponding to complete loss of exon 5. The exon 3-deleted variant (Δ3 isoform) lacks exon 3, resulting in partial loss of the succinate-Coenzyme Q (CoQ) oxidoreductase main activity region (14). In the exon 5-deleted variant (Δ5 isoform), the frameshift moves the stop codon to the 3′ non-coding region, adding an extra 70 aa to the final SDHC protein. However, frameshift mutations within this region abolish enzyme activity due to the loss of the heme binding region (13,14).

Certain SDH mutations cause the enzyme to become a significant source of mitochondrial superoxide production, which may contribute directly to disease progression (15). Of particular interest are diseases associated with reactive oxygen species (ROS) generated in the electron transport system (15,16). In the present study, to explore the mechanism of the PGL tumor development, the occurrence of S*DHC* gene ASVs was investigated, in particular, deleted exon 5 ASVs, which may induce frameshift mutations. The correlation between ROS and *SDHC* ASVs was also investigated.

## Materials and methods

### Cell culture

The HCT-15 (colorectal adenocarcinoma) cell line (Japanese Collection of Research Bioresources, Osaka, Japan) was maintained in Dulbecco’s modified Eagle’s medium (Gibco-BRL, Carlsbad, CA, USA) supplemented with 10% heat-inactivated fetal bovine serum under standard culture conditions (in a humidified atmosphere of 5% CO_2_ at 37°C), until cells reached 80–90% confluence. Cells were treated with Accumax (Innovative Cell Technologies, Inc., Logan, UT, USA) and counted using the Cell Lab Quanta SC flow cytometer (Beckman Coulter, Brea, CA, USA), according to the manufacturer’s instructions.

Cells were incubated for 2 h with or without 10 mM 2,2′-azobis(2-amidinopropane) dihydrochloride (AAPH), 1 mM H_2_O_2_ or 200 μM thallium trifluoroacetate (TTFA) (all Sigma-Aldrich, St. Louis, MO, USA). Apoptosis was determined by Annexin V (Beckman Coulter, Brea, CA, USA) and propidium iodide (Sigma-Aldrich) staining.

### Reverse transcription-polymerase chain reaction (RT-PCR) and expression vector generation

**Figure 1:**
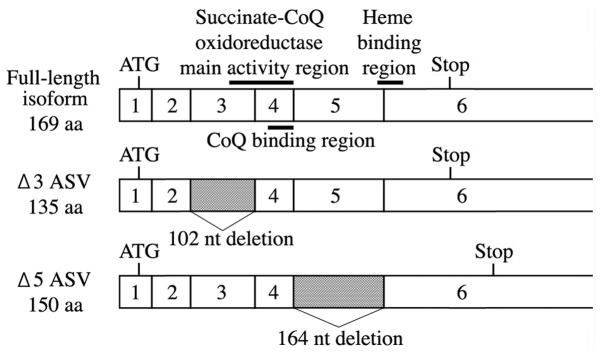
*SDHC* ASVs with deletions. Solid boxes indicate deleted exons and black bars indicate SDHC motif locations (specifically, succinate-CoQ oxidoreductase main activity, CoQ binding and heme binding regions). SDHC, succinate dehydrogenase complex, subunit C; ASV, alternative splicing variant; CoQ, coenzyme Q; aa, amino acid.

To identify *SDHC* gene ASVs, total RNA was extracted from normal lung tissue purchased from Clontech Laboratories (Mountain View, CA, USA). Total RNA from HTC-15 cells was extracted using the ReliaPrep™ RNA cell miniprep system (Promega Corporation, Madison, WI, USA), following the manufacturer’s instructions. To prepare cDNA, DNase-treated total RNA (0.1 μg) was incubated with M-MLV reverse transcriptase (Invitrogen Life Technologies, Carlsbad, CA, USA) and random primers (Invitrogen Life Technologies). The primer set for the amplification of *SDHC* cDNA was designed according to GenBank sequences: NM_003001.3 (full-length isoform), AB211234.1 (Δ3 ASV), and AB211235.1 (Δ5 ASV) (Table I; Fig. 1). PCR parameters were 95°C for 20 sec, 60°C for 30 sec and 45 cycles of 72°C for 20 sec, followed by a 10-min extension at 72°C, using AmpliTaq Gold DNA polymerase (Applied Biosystems, Foster City, CA, USA). PCR products were separated by electrophoresis on 2.0% agarose gels in Tris-borate-EDTA buffer, stained with ethidium bromide, and then detected under ultraviolet light. The PCR products were purified using the High Pure PCR Product purification kit (Roche Applied Science, Upper Bavaria, Germany), cloned into the pTriEX-3 neo expression vector (Merck Millipore, Darmstadt, Germany), and sequenced using the BigDye Terminator v3.1 Cycle sequencing kit (Applied Biosystems) with the ABI PRISM^®^ 3130 genetic analyzer (Applied Biosystems). Finally, sequences were compared with the full-length *SDHC* mRNA sequence. HCT-15 cells at densities of ~1.0–3.0×10^5^ were precultured with 2 mg of plasmid DNA and 3 ml of FuGENE HD transfection reagent (Promega Corporation). Following 15 min of incubation at room temperature, cells were incubated at 37°C and 5% CO_2_ for 24 h. *SDHC*-pTriEX-3 neo vector-transfected cells were selected using geneticin (Roche Applied Science).

### Real-time RT-PCR

Real-time PCR reaction mixtures were prepared using Kapa Sybr^®^ Fast qPCR Master Mix (Kapa Biosystems, Woburn, MA, USA). The primers used for the amplification of the full-length and ASV isoforms of *SDHC*, as well as the *GAPDH* mRNAs are shown in Table I. Real-time PCR reactions were performed for 45 cycles (95°C for 20 sec, 60°C for 25 sec and 72°C for 20 sec) using a real-time PCR system (iCycler iQ™ real-time detection system; Bio-Rad, Hercules, CA, USA). Following extension, an additional step was added whereby the sample was maintained at 75°C for 5 sec to allow for the fluorescence signal to be read. *GAPDH* housekeeping mRNA was amplified for normalization purposes, and mRNA expression levels are presented as the mRNA copy number per microgram of total RNA. Data are presented as the mean of three measurements.

### SDH activity

The transformed cells were pre-cultured at densities of ~1.0–3.0×10^5^ at 37°C and 5% CO_2_. The culture medium was removed and the cells were incubated for 1 h with 2.5 mM 3-(4,5-di-methylthiazol-2-yl)-2,5-diphenyltetrazolium bromide in 50 mM KPi (pH 8.0), 2 mM KCN and 350 mM 1-methoxy-5-methylphenazinium methylsulfate with 10 mM succinic acid. Following incubation, the reagent absorbance at 530 nm was measured using a FoodMark microplate reader (Bio-Rad). Data are presented as the mean of three measurements.

### Statistical analysis

All samples in the experiments were tested in triplicate or quadruplicate. All data are presented as the mean ± standard deviation. Differences between the mean values were evaluated using Student’s t-test. P<0.05 was considered to indicate a statistically significant difference.

## Results

### RT-PCR and real-time RT-PCR

**Figure 2:**
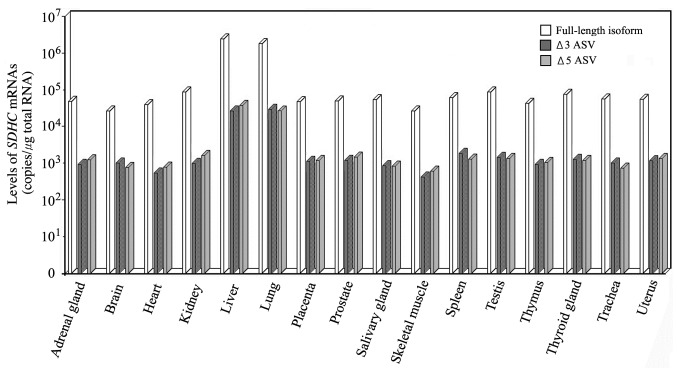
*SDHC* full-length and ASV mRNAs in human tissues. Left bars, full-length isoform; middle bars, Δ3 ASV; and right bars, Δ5 ASV. mRNA expression levels are presented as the mRNA copy number per microgram of total RNA. SDHC, succinate dehydrogenase complex, subunit C; ASV, alternative splicing variants.

Using RT-PCR with the primer sets for exons 1 and 6 of human *SDHC* mRNA, amplification products of three sizes were obtained; specifically, a major amplification product of 566 bp, and minor products of 464 and 402 bp (Fig. 1). To determine isoform expression *in vivo*, real-time RT-PCR was used to examine normal cells derived from 16 tissues, which included the adrenal gland, brain, heart, kidney, liver, lung, placenta, prostate, salivary gland, skeletal muscle, spleen, testis, thymus, thyroid gland, trachea and uterus. Full-length *SDHC* mRNA was expressed in every tissue examined. Δ3 and 5 ASV mRNAs were also ubiquitously expressed, but by two orders of magnitude lower than that of the full-length mRNA (Fig. 2).

**Figure 3:**
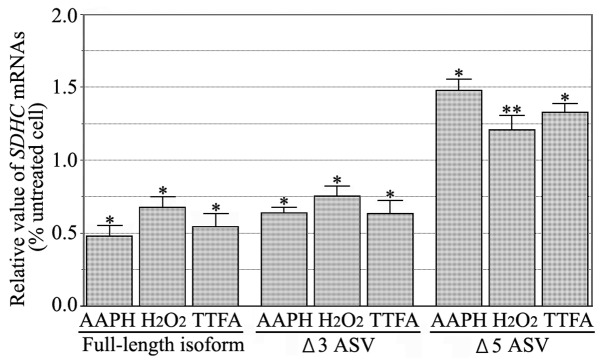
*SDHC* full-length and ASV mRNAs in HCT-15 cells treated with ROS reagents. To investigate mRNA dynamics following treatment with ROS reagents, quantification of *SDHC* mRNA was performed. mRNA expression levels are presented as the mRNA copy number per microgram of total RNA. ^*^P<0.05 and ^**^P<0.01, vs. untreated HCT-15 cells. SDHC, succinate dehydrogenase complex, subunit C; ASV, alternative splicing variants; ROS, reactive oxygen species; AAPH, 2,2′-azobis(2-amidinopropane) dihydrochloride; TTFA, thallium trifluoroacetate.

The treatment of HCT-15 cells with ROS reagents produced a detectable apoptosis signal, as determined by Annexin V. The incubation of HCT-15 cells with AAPH, H_2_O_2_ or TTFA exhibited no synchronization with the G_0_/G_1_ or G_2_/M phases of the cell cycle, suggesting that none of the reagents cause changes in state with regard to the cell cycle. The expression levels of *SDHC* ASV mRNAs following treatment with ROS reagents, were measured by real-time RT-PCR. The *SDHC* Δ5 ASV mRNA levels of the treated cells were marginally elevated compared with the untreated cells (P<0.05) (Fig. 3).

**Figure 4:**
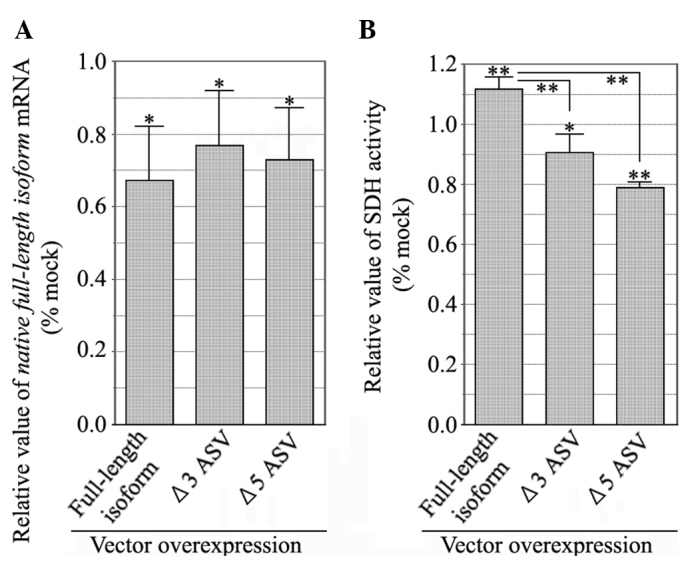
*SDHC* mRNA and SDH activity in HCT-15 cells treated with each overexpression vector. (A) Quantification of the endogenous full-length isoform mRNA in transfected cells. HCT-15 cells transfected with *SDHC*-pTriEX-3 neo vectors were selected with geneticin. (B) Quantification of SDH activity in transfected cells. ^*^P<0.05 and ^**^P<0.01, vs. the mock-transfected cells. Control, empty pTriEX-3 neo vector; full-length isoform, pTriEX-3 neo vector with *SDHC* full-length coding region; Δ3 isoform, pTriEX-3 neo vector with *SDHC* Δ3 coding region; and Δ5 isoform, pTriEX-3 neo vector with *SDHC* Δ5 coding region. SDH, succinate dehydrogenase; SDHC, SDH complex, subunit C; ASV, alternative splicing variant.

The expression levels of endogenous *SDHC* mRNA in *SDHC* ASV overexpressing cells were measured by real-time RT-PCR. Compared with the mock-transfected cells (transfected using empty pTriEX-3 neo vector), the endogenous *SDHC* mRNA levels in cells transfected with *SDHC* ASV overexpression vectors, were significantly decreased to ~30%. These results suggested that *SDHC* ASVs act as potent dominant-negative inhibitors of the full-length isoform (Fig. 4A).

### SDH activity

Full-length isoform-expressing cells significantly increased SDH activity, compared with the control cells expressing empty vector. However, cells overexpressing the Δ3 and Δ5 isoforms exhibited reduced SDH activity. Compared with SDH activity from full-length isoform-expressing cells, *SDHC* Δ5 ASV expression significantly decreased the SDH activity to ~40% (P<0.05) (Fig. 4B). These results corroborate the finding that *SDHC* ASVs act as potent dominant-negative inhibitors of the full-length isoform.

## Discussion

Genes involved in cellular respiration, such as *SDH*, are known to act as tumor suppressors, encoding proteins that inhibit tumor formation and cell proliferation (17,18). Mutations in tumor-suppressing genes promote carcinogenesis through DNA replication, causing events such as dysregulation in cell proliferation as well as the induction of apoptosis. Furthermore, cancer is a disease leading to tumor formation, and alternative splicing is triggered by disease onset. A number of mutations in human *SDH* genes are responsible for the development of PGLs, gastrointestinal stromal tumors and other types of cancer (5,18). In the current study, *SDHC* ASV was found to have a dominant-negative effect on SDHC activity, which provides a new possible target for cancer therapy. With improved understanding of the factors regulating *SDHC* alternative splicing, it may be possible to limit the tumor cell growth potential by promoting the production of non-functional or dominant-negative types of *SDHC* ASVs. During development, the dominant-negative function of the Δ5 variant may be significant when certain cells undergo differentiation, yet exhibit incomplete repression of *SDHC* transcription. During tumorigenesis, cancer cells may evolve from differentiated cell types, in which alternative splicing mechanisms may already be in place. Thus, *SDHC* alternative splicing in tumor cells may reflect pre-existing alternative splicing mechanisms present in the parent cells. In this study, the Δ5 isoform was not detected in high concentrations in the HCT-15 cell line protein extracts by western blot analysis (data not shown). As the stability of these variants is unknown, the Δ3 and 5 isoforms may not have been translated to sufficient levels to alter the SDHC activity. Therefore, the possibility that the Δ5 isoform causes dominant negative inhibition of the full-length isoform cannot be excluded. It appears likely that there is a more significant function for *SDHC* ASVs.

In cultured cells from *mev-1* mice expressing a mutated *SDHC* gene, mitochondrial ROS levels were increased (19,20). Essentially, the amount of active oxygen is tightly controlled by a balance between production and elimination *in vivo*. However, control failure of the *SDHC* gene induces the formation of tumors and cell death, due to a significant increase in the production rate of active oxygen (20). Similarly, in the current study, a partial correlation was observed between *SDHC* ASVs and ROS. This is the first example of active oxygen generated from an *SDHC* ASV, which directly affects cancer cell formation. ROS are widely known as ‘toxic factors’, which induce cellular toxicity and dysfunction through the oxidative damage of biomolecules. However, as a signal transduction factor, ROS are responsible for controlling cell differentiation, proliferation, cell death and cytoprotection (21,22). Therefore, it is imperative to balance the supporting ROS activities (a second messenger in intracellular signaling, as well as escape from excessive active oxygen species production) against their destructive behavior.

In the current study, to investigate the production of the superoxide anion (O_2_^−^), *SDHC*-overexpressing cells were stained with MitoSOX. Compared with the control cells (transfected with empty vector), Δ5 isoform-expressing cells produced excessive O_2_^−^ (data not shown). Therefore, although SDH activity is reduced in Δ5 isoform-overexpressing cells, the resulting metabolic byproduct generates excessive superoxide. The Δ5 isoform-overexpressing cells lack the heme binding region in the SDHC protein, and are unlikely to transfer electrons properly. Consequently, the accumulation and leakage of electrons may lead to coupling with nearby oxygen, subsequently generating excessive superoxide (23–25). In the present study, the Δ3 isoform did not generate excessive superoxide, which may be due to the incomplete inhibition of SDHC function, as the deletion of exon 3 only affects part of the succinate-CoQ oxidoreductase main activity region. Thus, expression of the Δ5 isoform is likely to preferentially cause superoxide production, leading to a variety of ROS. In turn, these ROS may react to oxidative stress genes, potentially altering the expression levels of cancer-related genes. Studies are currently underway to clarify the manner in which *SDHC* ASVs affect genes, leading to tumor formation. The results of this study provide a basis for more detailed future studies regarding the regulation of SDHC, and may lead to the development of clinical trials investigating SDHC and ROS-related diseases.

## Figures and Tables

**Table I:** Details of the primers used.

Gene	Accession no.	Deleted exon (nt)	Primer	Sequence	Product size, bp
Full-length isoform	NM_003001.3	-	Forward	5′-TATAGGTTCAAACCGTCCTCTG-3′	217
			Reverse	5′-GGATCAGTGCTGGACCTAAGC-3′	
Δ3 ASV	AB211234.1	Exon 3 (102)	Forward	5′-CTCAGCTCTGTATCAGAAATTGGT-3′	175
			Reverse	5′-TGCAAACTTAGCTGTGTGGATCAGTGC-3′	
Δ5 ASV	AB211235.1	Exon 5 (164)	Forward	5′-TATAGGTTCAAACCGTCCTCTG-3′	201
			Reverse	5′-TTCCTAGGTCCCACATCTGCA-3′	
GAPDH	X01677	-	Forward	5′-CGAGCCACATCGCTCAGACACC-3′	118
			Reverse	5′-GGCAACAATATCCACTTTACCAGAG-3′	

ASV, alternative splicing variant.
